# Sex Differences in Clinical Outcomes after Aortic Valve Intervention for Isolated Severe Aortic Stenosis

**DOI:** 10.3390/jcm12227025

**Published:** 2023-11-10

**Authors:** Teresa Sevilla, Noemí Ramos, Manuel Carnero, Ignacio J. Amat-Santos, Manuel Carrasco-Moraleja, Ana Revilla, Isidre Vilacosta, J. Alberto San Román

**Affiliations:** 1 Cardiology Department, Hospital Clínico Universitario de Valladolid, 47003 Valladolid, Spain; ijamat@gmail.com (I.J.A.-S.); manuelhcuv@gmail.com (M.C.-M.); arevillaorodea@gmail.com (A.R.); asanroman@secardiologia.es (J.A.S.R.); 2 Consorcio de Investigación Bioméidica en Red de Enfermedades Cardiovasculares, CIBER-CV, 28029 Madrid, Spain; 3 Cardiology Department, Hospital Clínico San Carlos, 28040 Madrid, Spain; noemi.rl92@gmail.com (N.R.); i.vilacosta@gmail.com (I.V.); 4 Cardiac Surgery Department, Hospital Clínico San Carlos, 28040 Madrid, Spain; mcarnero@me.com

**Keywords:** aortic stenosis, sex, long-term prognosis

## Abstract

There are known pathophysiologic and clinical differences according to sex in patients with aortic stenosis (AS). To evaluate if these differences persist after valve replacement, we conducted an observational study including 451 patients with symptomatic AS who survived aortic valve intervention (AVI) in two centers. Clinical data and mortality were evaluated at a mean follow-up of 5 years. 56% of patients were women. At baseline, women were older (80.6 vs. 78 years, *p* = 0.013), presented higher mean gradient (48 vs. 45 mmHg, *p* = 0.023), lower aortic valve area (0.70 vs. 0.74 cm^2^, *p* = 0.002) and higher systolic pulmonary artery pressure (36 vs. 33 mmHg, *p* = 0.016). They underwent percutaneous aortic valve replacement more frequently than men (47 vs. 35.9%, *p* = 0.017). At 5 years follow-up, women required more admissions due to heart failure (23 vs. 9%, *p* = 0.046) but they did not present higher cardiovascular nor overall mortality (27.7% vs. 29.8%, *p* = 0.741; 11.1 vs. 10.1%, *p* = 0.619, respectively). Female sex was an independent predictor of heart failure hospitalization at follow-up (HR 95% 1.16–4.22, *p* = 0.016). Women undergo AVI at a more advanced stage than men, resulting in a higher frequency of readmissions due to heart failure during the follow-up period, but not in higher mortality.

## 1. Introduction

Aortic stenosis (AS) is one of the most common valvular heart diseases in Western countries due to the increasing aging of their population [1]. Given the longer life expectancy in women, they represent a significant proportion of severe AS patients, which has led to great interest in the scientific community to identify whether there are specific gender differences regarding AS. 

While epidemiological studies do not demonstrate differences in the incidence of AS between sexes, distinctions have been identified in nearly all other aspects of the disease. From a morphological perspective, women have a smaller aortic annulus in accordance with their smaller body surface area (BSA), while men exhibit a three to four times higher prevalence of bicuspid aortic valve [2]. Physiopathologically, it has been demonstrated that men exhibit greater valve calcification, while women have a higher degree of valve fibrosis [3]. Additionally, there are differences in ventricular remodeling secondary to pressure overload. Most cardiac resonance studies show that, for the same degree of aortic stenosis, men develop greater myocardial mass, greater wall thickness, and larger ventricular volume [4,5]. However, it is not clear whether these morphological changes in the ventricle translate into a higher degree of fibrosis since studies looking for differences in myocardial fibrosis present conflicting results [4,5,6]. Regarding clinical presentation, women tend to be older and more fragile when they first experience symptoms. They also report dyspnea more frequently and are often in a more advanced functional class. In a consecutive cohort of 408 patients with severe AS referred to AVI, Fusch et al. report that 41% of women were in New York Heart Association (NYHA) functional class III or IV, compared to 18.9% of men [7]. The associated comorbidities are also different, with women having more hypertension, while men are more likely to be smokers and have a higher atherosclerotic burden [8]. 

There are many studies analyzing gender differences in prognosis following aortic valve replacement (AVI), either surgical (SAVR) or percutaneous (TAVR). In the short term, the majority of studies and meta-analyses agree that women have a higher rate of complications following the procedure, both with TAVR and surgery. Meta-analyses indicate that there are no sex differences in short-term mortality after TAVR but show a non-significant trend towards higher short-term mortality in women after SAVR [9,10]. Regarding long-term mortality, the evidence indicates that women have a worse prognosis than men after SAVR, while they exhibit greater survival after TAVR. Researchers attribute these differences to the lower degree of patient-prosthesis mismatch achieved with TAVR, a complication that is more relevant in women due to their smaller size. Additionally, they are also attributed to the more favorable outcome with TAVR in frail and older patients [9,10,11].

Most studies and meta-analyses show that women experience a higher degree of immediate complications after both types of procedures, SAVR and TAVR. Women also present a higher mortality after surgical replacement but not with TAVR, where long-term survival tends to be better due to their longer life expectancy [9,10,11]. Despite the extensive literature analyzing sex differences in AS, there is scarce evidence regarding the long-term symptomatic status of patients who undergo AVI and whether sex has an impact on the evolution of symptoms once the stenosis is relieved. With this in mind, we have conducted a study to investigate whether there are differences between both sexes in clinical manifestations and long-term prognosis following valve replacement.

## 2. Materials and Methods

**Figure 1:**
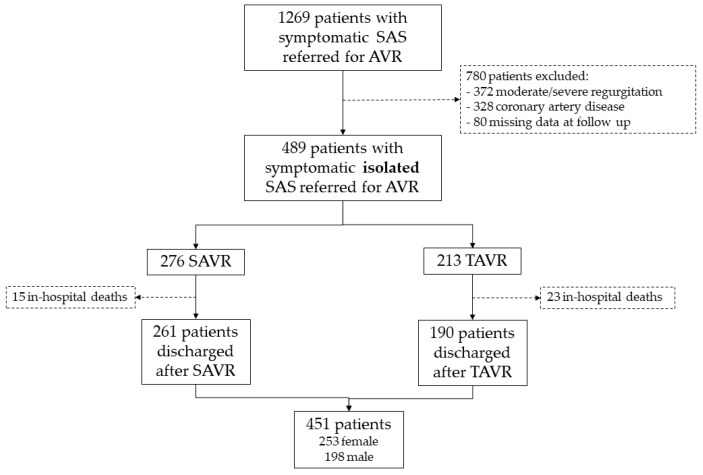
Patient inclusion flow chart. SAS: severe aortic stenosis; AVI: aortic valve intervention; SAVR: surgical aortic valve replacement; TAVR: transcatheter aortic valve replacement.

We conducted a two-center retrospective observational study on 451 consecutive patients referred for AVI due to symptomatic severe AS (aortic valve area ≤ 1 cm^2^ and/or mean gradient > 40 mm Hg) who were discharged from the hospital alive after the intervention between 2013 and 2017. We excluded patients with concurrent moderate or severe valvular regurgitation and significant coronary stenosis on coronary angiography. Both centers are tertiary referral hospitals for cardiology and cardiovascular surgery. Figure 1 shows the flow chart of the patients included.

The median follow-up duration was 56 months (IQR: 40–73). The follow-up was conducted through in-person visits at the intervention center. In cases where in-person visits were not feasible, electronic medical records were reviewed, direct contact was established with the patient’s responsible physician or the patient was contacted by phone to collect necessary follow-up data. The study endpoints were the overall all-cause mortality and hospitalization for heart failure, defined as any event requiring hospital admission and intravenous diuretic therapy administration. 

Statistical analysis: categorical variables are presented as frequencies, and comparisons between groups were performed using the χ^2^ or Fisher’s exact test when necessary. Continuous variables are expressed as median (25th–75th IQR). The normal distribution of continuous variables was tested using the Kolmogorov–Smirnov test. Comparisons between groups were performed using Mann–Whitney U. We identified that women had higher rates of admission due to heart failure at follow-up. To assess the influence of sex on heart failure admission, we performed an explanatory multivariable Cox proportional hazard regression with Fine and Gray competing risks using mortality as a competing event and reports estimations as subdistribution hazard ratios (sHR). Kaplan–Meier tests in the absence of competing risk and adjusted cumulative incidence function (CIF) were performed to show the effect on this variable. Possible collinearity among the different introduced variables was evaluated and controlled for overfitting in the model. Verification of proportional hazard assumption was performed by Schoenfeld residuals. All tests were two-sided at the 0.05 significance level. The errors and confidence intervals for the models were calculated using 500 bootstrap samples. All analyses were performed using R software, V.3.6.1 (R Project for Statistical Computing).

## 3. Results

Table 1 shows the comparison between males and females. At baseline, we identified some epidemiological differences between the two sexes. Women were older and more frequently hypertensive, while men were significantly more likely to be current or former smokers. Additionally, men had higher rates of chronic obstructive pulmonary disease and peripheral arterial disease. We did not identify differences in the proportion of diabetic patients, those with renal insufficiency, or those with atrial fibrillation between the two groups. The clinical manifestations also showed marked differences based on gender. Angina and syncope were significantly more frequent among men. Women exhibited a more advanced dyspnea functional class, although the differences did not reach statistical significance (functional class III–IV 45% vs. 36.5%, *p* = 0.067). In the baseline echocardiogram, women showed signs of more advanced valvular disease, with a higher mean gradient, a smaller aortic valve area, and higher pulmonary artery systolic pressure values. The type of intervention was TAVR in 47% of women, while only in 35.9% of men, *p* = 0.017. There were no significant differences in the prescribed treatment between the two sexes. At discharge, 43% of the patients were prescribed ACE inhibitors, 58.3% beta-blockers, and up to 71% received diuretic treatment. At that time, neither neprilysin inhibitors nor sodium-glucose co-transporter 2 inhibitors were part of the therapeutic options. Therefore, it can be considered that the cohort had a fairly optimized treatment for heart failure, as patients with an ejection fraction below 45% were less than 10%. Table 2 shows these data.

During a 5-year follow-up period, 57% of the patients presented cardiac symptoms after AVI. There were no differences between men and women in the frequency of symptom occurrence. In contrast to what was observed before the intervention, there were no differences in the types of symptoms reported between the two groups. The overall mortality was 28.6%, with cardiovascular mortality at 10.6%, and there were no differences between genders. However, women required hospitalization due to heart failure significantly more often than men (18.6% vs. 10.1%, *p* = 0.012). No relevant differences were identified between the two centers. 

### Predictors of Heart Failure Hospitalization during Follow-Up

**Figure 2:**
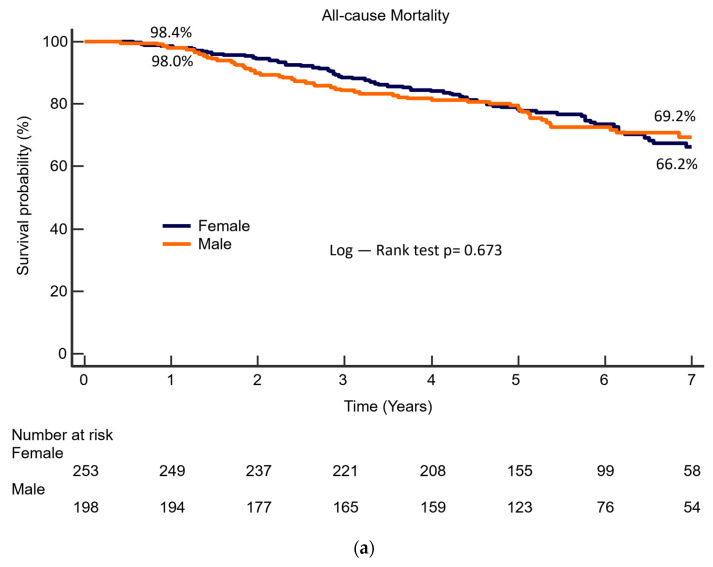

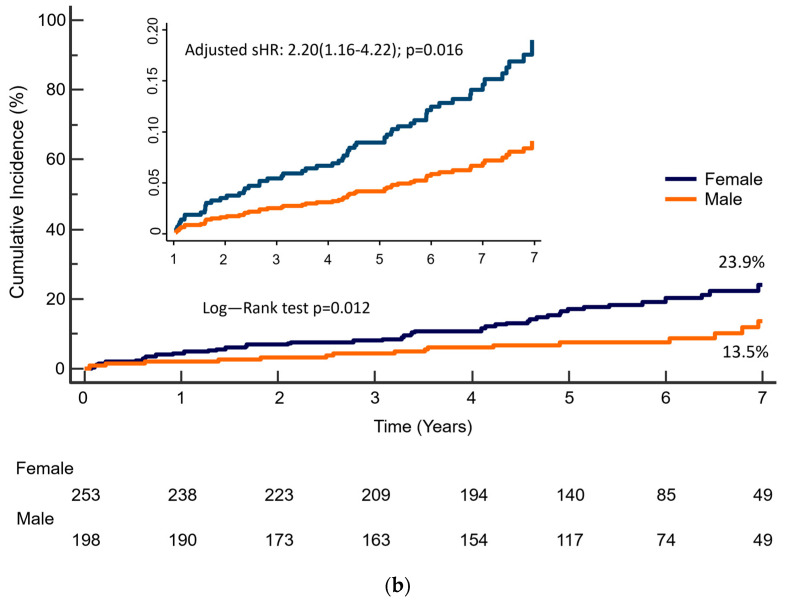
(**a**) Kaplan–Meier survival curve for all-cause mortality after AVI, according to sex. (**b**) Kaplan-Meier and adjusted cumulative incidence function for heart failure rehospitalization after AVI, according to sex.

To better characterize the role of gender in the development of heart failure during follow-up, we conducted a multivariate analysis to identify independent predictors of hospitalization due to heart failure. Table 3 presents the results of this analysis. Female sex is independently associated with hospitalization for heart failure during follow-up (HR 95% CI: 1.16–4.22, *p* = 0.016), as are age, prior heart failure, body mass index, left ventricular end-diastolic diameter, and a history of atrial fibrillation. The type of intervention (TAVR vs. SAVR) is not independently related to the development of heart failure during follow-up. Figure 2 shows the Kaplan–Meier curve for long-term survival (Figure 2a) and adjusted cumulative incidence estimates for heart failure rehospitalization after AVI (Figure 2b), according to sex.

## 4. Discussion

This study follows a contemporary cohort of patients with isolated symptomatic AS referred for AVI to evaluate if there are gender differences in long-term clinical status and prognosis after the intervention. We have found many differences between both sexes at the time of referral: women who undergo AVI are older, present a more advanced valve disease and they show a non-significant tendency to present a more advanced functional class. Men, instead, have more associated comorbidities and complain of syncope and angina more often. These epidemiological and clinical differences are consistent with previous evidence [7].

It has been described that men have a higher indexed myocardial mass, larger ventricular volumes, and a worse ejection fraction for the same degree of stenosis than women [4,6,12,13,14]. We have also identified that men have larger end-diastolic diameters and greater posterior wall thickness, but we have not found differences in ejection fraction between men and women. Although extensively described, the clinical significance of the differences in ventricular volumes and ventricular mass is likely limited, especially when considering that healthy men have higher left ventricular mass and ventricular volumes than women, even when indexed by BSA [15]. Regarding the ejection fraction, all studies reporting a lower ejection fraction in men did not exclude patients with confirmed coronary artery disease. In fact, Petrov et al. reported a series of isolated aortic stenosis cases without associated coronary disease, in which, similar to our series, there were no statistically significant differences in ejection fraction between the two sexes [16].

We have identified clear differences in referral patterns for intervention. At the time of referral, women present higher gradients, smaller aortic valve areas, and higher pulmonary artery pressure. This contrasts with prior evidence based on observational imaging studies, which suggests that women develop symptoms earlier [6] and exhibit lower gradient values and smaller valve areas than men, in part due to their smaller body surface area [12]. The differences we have found are similar to other series obtained at the time of valve replacement [7,14] and suggest that women are referred for valve replacement at a more advanced stage of the disease compared to men. In our series women undergo TAVR more frequently than men. This pattern has been extensively described and it is considered to be multifactorial: women are older, present a smaller body surface area, have higher frailty, and tend to present better outcomes after TAVR [14,17,18]. Moreover, for most of the time when patients in this cohort were referred for AVI, TAVR was recommended mostly for symptomatic AS patients who were deemed unfit for SAVR by the Heart Team. The majority of these patients were elderly, as are the women in this cohort. 

Previous studies analyzing sex differences in outcomes after AVI have focused on major end-points such as mortality, cardiovascular mortality, myocardial infarction, or stroke. Observational data have reported heterogeneous outcomes after SAVR [7,18,19,20], but metanalysis shows that women present higher mortality rates compared to men following SAVR [10]. Regarding TAVR, there is a consensus that despite a higher rate of procedural complications, women show a better long-term prognosis after TAVR [9,10,11,17,21,22]. In our series, women undergo TAVR more frequently than men, and we have found no differences in long-term mortality between sexes (female vs. male: 27.7% vs. 29.8%, *p* = 0.619). There is limited knowledge about the differences in symptoms and quality of life after AVI. We have identified that there are no differences in the clinical status after the intervention. The persistence of symptoms is high in both genders but, unlike our baseline observations, there are no differences between sexes in the reported symptoms. However, we have identified that women are admitted due to heart failure more often than men (18.6% vs. 10.1%, *p* = 0.012). This may be attributed to the older age of women in the series and their interventions at a more advanced stage, with higher pulmonary pressure values. Female sex was independently associated with heart failure admission in the multivariate analysis. Furthermore, the presence of dyspnea before intervention was already more frequent in women than men in our series. This might be related to diastolic dysfunction, ventricular fibrosis, or irreversible remodeling, factors that would favor heart failure in the follow-up, suggesting that those patients should have been intervened upon earlier.

Despite similar long-term survival rates, the higher rate of hospitalization for heart failure in women is a significant finding. Time spent in the hospital is a major determinant of the quality of life of elder patients and quality of life should be considered one of the primary objectives in patient care, especially in those with a limited life expectancy. In fact, the persistence of symptoms after AVI has been identified as a predictor of patients with poor outcomes [23].

Our study has some limitations. Firstly, it is a retrospective registry, so despite achieving consecutive inclusion, there is a risk of selection bias, as well as challenges in controlling confounding variables and limitations in result generalization. The selection of the endpoints was also retrospective, although mortality and heart failure hospitalization are robust and well-established hard endpoints used in many other studies. Additionally, we have included only patients with isolated aortic stenosis, which is both a strength and a limitation. On one hand, it represents a very homogeneous cohort that helps us better understand the consequences of aortic stenosis per se, but on the other hand, it deviates from the real patient who often presents with coronary disease or other valvular disease.

## 5. Conclusions

Compared to men, women who survive AVI for severe AS are older, have more severe valve disease, and undergo TAVR more frequently. In follow-up, they do not exhibit higher mortality than men, but they do have a higher need for hospitalization due to heart failure. Our results should encourage further research focused on the impact of sex on the long-term outcomes of AS.

## Figures and Tables

**Table 1:** Clinical, echocardiographic, and follow-up data of the cohort.

	Total Population (*n* = 451)	Female (*n* = 253)	Male (*n* = 198)	*p*-Value
**Clinical characteristics**				
Age—years	79.7 [72.0–84.6]	80.6 [73.4–85.0]	78.0 [70.4–84.0]	**0.013**
TAVR—*n* (%)	190 (42.1)	119 (47.0)	71 (35.9)	**0.017**
BMI	27.8 [25.3–30.9]	27.5 [25.0–30.8]	28.2 [25.8–30.8]	0.174
BSA	1.74 [1.62–1.87]	1.66 [1.54–1.76]	1.85 [1.75–1.95]	**<0.001**
Smoker—*n* (%)				
No	337 (74.7)	233 (92.1)	104 (52.5)	**<0.001**
Yes	25 (5.5)	3 (1.2)	22 (11.1)	
Former	89 (19.7)	17 (6.7)	72 (36.4)	
Hypertension—*n* (%)	334 (74.1)	198 (78.3)	136 (68.7)	**0.021**
Dyslipemia—*n* (%)	269 (59.6)	154 (60.9)	115 (58.4)	0.592
Diabetes mellitus—*n* (%)	138 (30.6)	71 (28.1)	67 (33.8)	0.187
Peripheral vascular disease—*n* (%)	22 (4.9)	7 (2.8)	15 (7.6)	**0.018**
Atrial fibrillation—*n* (%)	124 (27.5)	68 (27.3)	56 (29.0)	0.692
COPD—*n* (%)	50 (11.1)	13 (5.1)	37 (18.7)	**<0.001**
Chronic kidney disease—*n* (%)	89 (19.7)	54 (21.3)	35 (17.7)	0.332
Previous Stroke/TIA—*n* (%)	34 (7.5)	20 (7.9)	14 (7.1)	0.730
Dyspnea on effort—*n* (%)	419 (92.9)	238 (94.1)	181 (91.4)	0.275
Heart Failure—*n* (%)	131 (29.0)	78 (30.8)	53 (26.8)	0.346
NYHA functional class—*n* (%)				
I	6 (1.4)	4 (1.7)	2 (1.1)	0.134
II	239 (57.0)	126 (52.9)	113 (62.4)	
III	162 (38.7)	101 (42.4)	61 (33.7)	
IV	12 (2.9)	7 (2.9)	5 (2.8)	
NYHA functional class III–IV—*n* (%)	174 (41.6)	108 (45.4)	66 (36.5)	0.067
Angina—*n* (%)	129 (28.6)	60 (23.7)	69 (34.8)	**0.009**
CCS class—*n* (%)				
1	34 (26.4)	13 (21.7)	21 (30.4)	0.321
2	83 (64.3)	41 (68.3)	42 (60.9)	
3	12 (9.3)	6 (10.0)	6 (8.7)	
Syncope—*n* (%)	59 (13.1)	26 (10.3)	33 (16.7)	**0.046**
Type of syncope—*n* (%)				
At rest	24 (40.7)	13 (50.0)	11 (33.3)	0.999
On Exercise	32 (54.2)	12 (46.2)	20 (60.6)	
Unknown	3 (5.1)	1 (3.8)	2 (6.1)	
**Echocardiographic parameters**				
Mean aortic gradient—mmHg	47.0 [40.0–57.0]	48.0 [40.0–58.5]	45.0 [40.0–55.0]	**0.023**
Max Velocity—m/seg	4.40 [4.00–4.80]	4.46 [4.09–4.85]	4.36 [4.03–4.80]	0.123
Valve area—cm^2^	0.70 [0.60–0.90]	0.70 [0.54–0.85]	0.74 [0.60–0.94]	**0.002**
AVA index cm^2^/m^2^	0.41 [0.32–0.51]	0.41 [0.32–0.52]	0.40 [0.32–0.50]	0.380
LVEF—%	60.0 [58.0–65.0]	62.0 [59.0–66.0]	60.0 [56.0–65.0]	0.106
LVEF < 45%	42 (9.3)	21 (8.3)	21 (10.6)	0.403
EDLVD—mm	46 [42.0–50.0]	45.0 [41.0–50.0]	47.0 [43.0–51.0]	**<0.001**
ESLVD—mm	29.0 [25.0–33.0]	28.0 [24.0–32.0]	30.0 [27.0–34.0]	**<0.001**
IVS—mm	14.0 [13.0–16.0]	14.0 [13.0–16.0]	14.0 [13.0–17.0]	0.126
PW—mm	12.0 [11.0–14.0]	12.0 [10.0–13.0]	12.0 [11.0–14.0]	**0.009**
PASP—mmHg	33.0 [25.0–45.0]	36.0 [25.0–47.0]	33.0 [25.0–42.5]	**0.016**
TAPSE—mm	20.0 [18.0–22.0]	20.0 [18.0–22.0]	20.0 [19.0–23.0]	0.066
**Blood analysis**				
Hemoglobin—g/dL	13.1 [11.8–14.1]	12.8 [11.6–13.6]	13.6 [12.0–14.7]	**<0.001**
Creatinine—μmol/L	0.97 [0.80–1.20]	0.89 [0.70–1.11]	1.04 [0.90–1.29]	**<0.001**
**Symptoms at Follow-up**				
Any symptoms at Follow-up	259 (57.4)	151 (59.7)	108 (54.5)	0.273
Dyspnea—*n* (%)	253 (56.1)	149 (58.9)	104 (52.5)	0.176
Angina—*n* (%)	9 (2.0)	4 (1.6)	5 (2.5)	0.515
Syncope—*n* (%)	19 (4.2)	12 (4.7)	7 (3.5)	0.526
Death—*n* (%)	129 (28.6)	70 (27.7)	59 (29.8)	0.619
Cardiovascular death—*n* (%)	48 (10.6)	28 (11.1)	20 (10.1)	0.741
Heart Failure Hospitalization—*n* (%)	67 (14.8)	47 (18.6)	20 (10.1)	**0.012**

BMI: body mass index. BSA: body surface area. EDLVD: end-diastolic left ventricular diameter; ESLVD: end-systolic left ventricular diameter; IVS: interventricular septum; PW: posterior wall; LVEF: left ventricular ejection fraction; PASP: pulmonary artery systolic pressure; TAPSE: tricuspid annular plane systolic excursion; TIA: transient ischemic attack.

**Table 2:** Medication prescribed at discharge.

Medication Discharge	N	Total Population (*n* = 451)	Female (*n* = 253)	Male (*n* = 198)	*p*-Value
ASA—*n* (%)	337	206 (57.4)	109 (58.0)	97 (56.7)	0.810
Clopidogrel—*n* (%)	359	56 (16.2)	33 (18.2)	23 (13.9)	0.279
Warfarin—*n* (%)	370	193 (52.2)	104 (52.3)	89 (52.0)	0.967
DOACs—*n* (%)	282	13 (4.6)	7 (4.8)	6 (4.4)	0.878
ACE inhibitors—*n* (%)	336	145 (43.2)	73 (42.0)	72 (44.4)	0.645
Diuretics—*n* (%)	336	240 (71.4)	131 (75.3)	109 (67.3)	0.105
Betablockers—*n* (%)	336	196 (58.3)	106 (60.9)	90 (55.6)	0.319
Nitrates—*n* (%)	331	1 (0.3)	0 (0.0)	1 (0.6)	0.477
Statins—*n* (%)	315	190 (60.3)	102 (62.2)	88 (58.3)	0.478
Calcium antagonist—*n* (%)	307	31 (10.1)	18 (11.2)	13 (8.9)	0.509

ASA: acetylsalicylic acid. DOACs: direct oral anticoagulants. ACE: angiotensin-converting enzyme.

**Table 3:** Univariate and multivariate competing risk analysis of predictors of heart failure admission at follow-up.

	Univariate Analysis				Multivariate Analysis			
	Sub-Hazard Ratio	Bootstrap 95.0% CI		*p*-Value	Sub-Hazard Ratio	Bootstrap 95.0% CI		*p*-Value
		Lower	Upper			Lower	Upper	
**Tavi Intervention**	1.87	1.20	2.92	** 0.005 **				
**Female**	2.00	1.17	3.44	** 0.012 **	2.20	1.16	4.22	** 0.016 **
**Age**	1.05	1.02	1.08	** 0.002 **	1.04	1.01	1.07	** 0.018 **
**Previous Heart Failure**	2.51	1.72	3.65	** <0.001 **	1.77	1.05	2.96	** 0.031 **
**BMI**	1.04	1.00	1.09	** 0.035 **				
**Previous AF**	2.92	1.79	4.77	** <0.001 **	2.99	1.93	4.65	** <0.001 **
**Baseline EDLVD**	1.03	1.01	1.06	** 0.013 **	1.04	1.01	1.08	** 0.014 **
**LVEF**	0.98	0.96	1.00	0.107				

BMI: body mass index; EDLVD: end-diastolic left ventricular diameter; LVEF: left ventricular ejection fraction.

## Data Availability

The data presented in this study are available on request from the corresponding author. The data are not publicly available due to privacy aspects.
